# Supplementary material for: Comprehensive Analysis of Differentially Expressed Unigenes under NaCl Stress in Flax (Linum usitatissimum L.) Using RNA-Seq
Source: Int J Mol Sci. 2019 Jan 16;20(2):369. doi: 10.3390/ijms20020369 (PMC6359340; doi:10.3390/ijms20020369)
Supplement: Supplementary file 1 [file ijms-20-00369-s001.zip › ijms-410872 Supplementary material for final/Supplementary Files/New Microsoft Word Document.pdf]

Table S1. The regulated unigenes in different periods. C-vs-N1, C-vs-N2 and C-vs-N4 representing the DEUs under the exposure time of 12h, 24h and 48h in NaCl solution, respectively. Common means the common genes in different periods.

Table S2. Annotation of the co-expressed unigenes in flax. The DEUs were annotated in the public database of Pfam, KOG, KEGG, KO and GO; The annotated results with *Arabidopsis thaliana* genome list in Best-hit arabi, Arabi-symbol and Arabi-defline.

Table S3. The developed EST-SSRs with primer pairs.

Table S4. Primer sequences for qRT-PCR. AY857865, is the *L. ussitatissimum Act1* gene, *here* as the internal reference gene.
